# EFFECTS OF RENAL DYSFUNCTION ON HEALING OF COLONIC ANASTOMOSIS: EXPERIMENTAL STUDY IN WISTAR RATS

**DOI:** 10.1590/0102-672020180001e1398

**Published:** 2018-12-06

**Authors:** Carlos Eduardo da SILVA, João Carlos Domingues REPKA, Carlos José Franco de SOUZA, Jorge Eduardo Fouto MATIAS

**Affiliations:** 1 Experimental Research Laboratory, Maternity Hospital Angelina Caron, Campina Grande do Sul, PR;; 2 Program of Post-Graduation in Surgical Clinic of the Federal University of Paraná, Curitiba, PR, Brazil

**Keywords:** Nephrectomy, Uremia, Wound healing, Colon, Rats Wistar, Nefrectomia, Uremia, Cicatrização, Cólon, Ratos Wistar

## Abstract

**Background:**

Chronic kidney disease affects more than 500 million people worldwide. In this context, the uremic toxins present are related to worsening in tissue healing.

**Aim:**

Evaluate on healing of colonic anastomosis in uremic rats, serum and anatomopathological indicators, which may be related to the change tissue repair process.

**Methods:**

Twenty Wistar rats, were randomly separated into two groups. In the sham group they were submitted to 5/6 nephrectomy simulation in left kidney, simulation right nephrectomy, median laparotomy, colotomy and colorraphy. In the uremia group, they were submitted to 5/6 nephrectomy of the left kidney, total nephrectomy of the right kidney and median laparotomy, colotomy and colorraphy. Were collected for serum urea, creatinine and CRP dosages and the colonic segments were studied for evaluation of granulation tissue, collagen maturation, microvascular and myofibroblasts density, and cell viability. Through histochemical processing, microvascular density was evaluated by anti-CD34 monoclonal antibody marking, cell viability by cell proliferation nuclear antigen screening and myofibroblasts density with monoclonal anti-α-actin antibody. Computerized histometry was used for evaluations of collagens type I and III by the coloration of picrosirius.

**Results:**

The group submitted to nephrectomy 5/6, compared to the sham group, show urea increase (p<0.0000) and higher C reactive protein (p=0.0142). Decrease of granulation tissue formation (border reepithelialization p=0,0196, angiofibroblast proliferation p=0.0379), mean collagen I (p=0,0009) and collagen III (p=0,016), microvascular density (p=0,0074), cell proliferation nuclear antigen (p<0,0000) and myofibroblasts (p<0,0001).

**Conclusion:**

The uremia induced by nephrectomy 5/6 model establishes negative impact in the colonic wound healing.

## RESUMO

**Racional:**

A doença renal crônica atinge mais de 500 milhões de pessoas em todo o mundo. Neste contexto, as toxinas urêmicas estão relacionadas ao comprometimento da cicatrização tecidual.

**Objetivo:**

Avaliar, na cicatrização de anastomoses colônicas de ratos urêmicos indicadores séricos e anatomopatológicos que possam estar relacionados com alteração do processo de reparação tissular.

**Métodos:**

Utilizaram-se 20 ratos Wistar divididos aleatoriamente em dois grupos. No grupo simulação eles foram submetidos à simulação da nefrectomia 5/6 do rim esquerdo, simulação de nefrectomia total do rim direito, laparotomia mediana, colotomia e colorrafia. No grupo uremia, eles foram submetidos à nefrectomia 5/6 do rim esquerdo, nefrectomia total do rim direito, laparotomia mediana, colotomia e colorrafia. Coletaram-se amostras de sangue para dosagens séricas da ureia, creatinina e proteína C reativa, e do cólon para processamentos histológicos e histoquímicos na avaliação do tecido de granulação, maturação de colágeno, densidade microvascular e de miofibroblastos, viabilidade celular cicatricial. Empregou-se a histometria computadorizada para as avaliações de colágenos tipos I e III, densidade microvascular pela marcação com anticorpo monoclonal anti-CD34, viabilidade celular pela pesquisa do antígeno nuclear de proliferação celular e a densidade de miofibroblastos com anticorpo monoclonal anti-α-actina.

**Resultados:**

O grupo submetido à nefrectomia 5/6, em comparação ao grupo simulação, demonstraram aumentos da ureia sérica (p<0,0000) e proteína C reativa (p=0,0142), redução da formação de tecido de granulação (reepitelização de bordas p=0,0196, proliferação angiofibroblástica p=0,0379), porcentagens de colágeno I (p=0,0009) e colágeno III (p=0,016), densidade microvascular (p=0,0074) e miofibroblastos (p<0,0001) e antígeno nuclear de proliferação celular (p<0,0000).

**Conclusão:**

A uremia induzida pelo modelo de nefrectomia 5/6 determina impacto negativo no processo de cicatrização colônico.

## INTRODUCTION

Healing is a complex process, which has begun to be understood to a greater extent in recent years. However, the knowledge thereof must still be extended in view of the innovative preventive and curative measures available to surgeons, thus reducing the possibility of complications in the handling of patients who need surgical aggression to cure their ills^24^ ^,^ ^28^.

Since cicatrization is developed by a harmonic set of local cellular and biochemical events, common to several sectors of the organism, it can be said that these influence its basic intermediary mechanisms such as hemostasis, inflammation, cell proliferation and wound remodeling.

In this context, uremic toxins, generated in renal dysfunction, are responsible for the progression of chronic renal disease (CKD) by inducing loss of residual renal function, triggering systemic and vascular inflammatory responses and thus, increasing renal endothelial dysfunction. Uremic toxins are responsible for the progression of CKD and loss of residual renal function; however, no specific time points to the onset of uremia in patients with progressive loss of renal function^19^. Other adverse effects of CKD include decreased phagocytic activity of polymorphonuclear cells, impaired tissue healing, delayed cicatricial inflammatory process, low proliferation of fibroblasts and endothelial cells, low tissue levels of hydroxyproline and collagen, subcutaneous connective tissue and granulation tissue^30^.

Among surgical procedures, gastrointestinal operations are among the most frequently performed. In patients with CKD when they need some intestinal surgical approach, even under uremic conditions, this will be the only decision to be made by the surgeon in the search for a solution for the patient, as it occurs in emergency situations or in cases of renopancreatic transplantation. Among the gastrointestinal surgical complications, the most described are the failures in anastomotic healing represented by 3.4% to 12% of dehiscence, with the main cause being the metabolic disorders of uremia secondary to CKD^16^.

Although a large number of scientific information on the surgical induction of renal dysfunction in rats is available, there are still few studies on the effects of uremia on intestinal healing. Animal models of renal dysfunction approach the human condition and are important for the understanding of the disease and for the development of new therapeutic strategies^3^ ^,^ ^4^.

Therefore, the objective of the present study was to evaluate, in an experimental model of uremia in rats, specific serum and anatomopathological aspects in the healing of colonic anastomosis.

## METHOD

Twenty rats (*Rattus norvegicus albinus*, Rodentia mammalia) of the Wistar lineage, with ages between 143-152 days and weights of 249.2±13.80 g were used. They were separated into two groups.

### Surgical procedure

Anesthesia was given in two stages. Firstly, they were submitted to sedation by inhalation and after intramuscular inoculation in both posterior calves of the anesthetics ketamine hydrochloride, associated with xilasin hydrochloride.

In order to induce uremia in the animals of the uremia group, the surgical procedure called nephrectomy 5/6, described by Viana et al.^33^ was used, which consists of the following steps: partial nephrectomy, when both renal poles are resected, and seven days after complete nephrectomy of the contralateral kidney. In D0, partial nephrectomy was performed by means of a left lumbar incision of about 3 cm in extension and the peritoneal cavity was accessed for the exposure of the left kidney that was drawn out of the cavity, decapsulated preserving the gland adrenal axis and ablation of the renal poles with Argon plasma electrocoagulator (Argon 4 - WEN ®) corresponding to approximately 2/3 renal mass. The thread was preserved, as well as the vascularization and the ureters.

In the simulation group, only a 4 cm extension incision was made and the peritoneal cavity was accessed with exposure of the kidney, which was drawn out. In both groups, wall closure was done by continuous suturing with monofilament nylon 3.0 wire in musculoaponeurotic and cutaneous planes.

### Sample collection

On the 7^th^ day of post-colotomy and colorraphy (D21) evolution, the rats were again weighed on analytical balance and underwent closed-loop halothane inhalation sedation and anesthetized by intramuscular injection of ketamine hydrochloride.

Cardiac puncture was then performed with the collection of 8-10 ml of blood, which corresponded to an exanguinative puncture and induction of cardiorespiratory arrest. Blood samples were immediately sent to the laboratory for serum levels of urea, creatinine, and C-reactive protein.

Also under anesthesia and with evidence of death, the abdominal cavity was extensively opened, an inventory of the cavity was made, the colonic segment was located, which was incised and rayed, resected, extended on filter paper, washed with phosphate buffered saline solution (PBS) pH 7,4 and fixed in formalin.

### Evaluations

To evaluate the evolution, initial and periodic weighing were used on days D0, D2, D4, D7, D9, D12, D14, D17 and D19, as well as the observation of the animals evaluating the search for food, water and ambulation as indicators of normality in comparison between groups. Evidence of uremic state induction was made by serum urea and creatinine at the end of the study period, using an automated method with specific reagents for urea, creatinine and C-reactive protein^6^ ^,^ ^25^.

Microscopic evaluations of the healing process of the colonic anastomosis were performed through tissue granulation analysis, formation of collagens types I and III, microvascular density, cell proliferation and myofibroblasts density.

Sections were made perpendicular to the largest axis of the suture, in triplicates for each of the histological determinations, with a microtome 4 µm thick and fixed in slides to be stained according to the evaluation to be made. The formation of granulation tissue by H&E staining was evaluated by microscopy^31^. Was used computerized histometry for the analysis of collagen types I and III by Picrosirius^18^, microvascular density by anti-CD34^15,23^ monoclonal antibody labeling, cell viability by nuclear proliferation cell antigen^5^ ^,^ ^15^ and myofibroblasts density with anti-α-actin monoclonal antibody^15^ ^,^ ^21^.

### Statistical analysis

The results were expressed as mean±standard deviation and the ANOVA and Student T tests were used with p<0.05 for comparisons between groups using the GraphPad InStat software.

## RESULTS

### Weight evaluation

The rats of the uremia group presented greater weight loss during the experiment, but without statistical differences in the weighing moments.

### Biochemical evaluations

#### *Dosages of urea*

**FIGURE 1:**
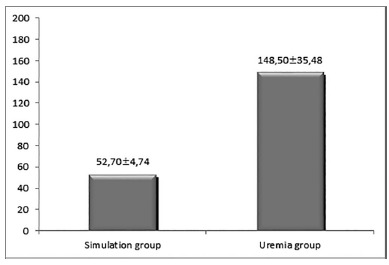
Graph showing the arithmetic means of the urea (mg/ml) and creatinine (mg/ml) dosages

The model employed was able to induce uremia in the experimental group in relation to the simulation group (p<0.0000), although the higher creatinine levels in the uremia group were not significantly different (p=0.0904) simulation group (Figure 1).

#### *C-reactive protein dosages*

**FIGURE 2:**
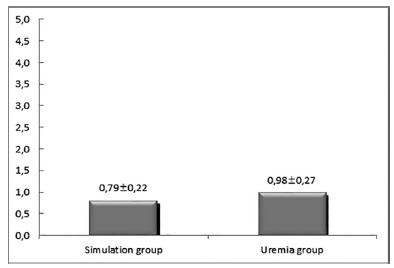
Graph showing the arithmetic means of the dosages of ultra-sensitive C reactive protein (mg/ml)

As shown in Figure 2, the rats in the uremia group had significantly higher C-reactive protein values than the simulation group (p=0.0142)

### Microscopic evaluations

#### *Histopathological evaluation of granulation tissue*

In the evaluation of the colonic cicatrization process, the superiority of the simulation group over the uremia group was clearly observed, with a statistically significant impact on reepithelialization of borders (p=0.0196) and angiofibroblast proliferation (p=0.0379).

#### *Histometric evaluation of the percentage of collagen types I and III*

**FIGURE 3:**
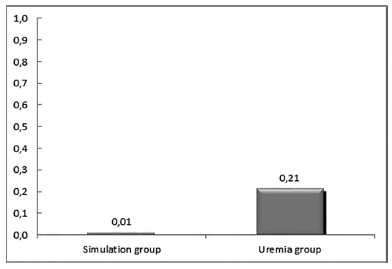
Graph showing the arithmetic means of the percentages of collagens type I and III in the intestinal scar tissue

As shown in Figure 3, the rats in the uremia group presented significantly worse results than the simulation group in the mean percentages of collagen I (p=0.0009) and in relation to collagen III (p=0.016).

#### *Microvascular density evaluation*

**FIGURE 4:**
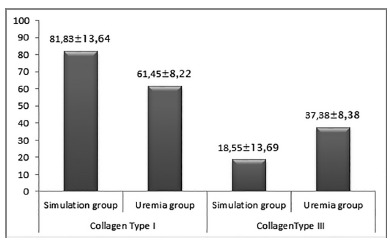
Graph showing the arithmetic means of the microvascular density quantification in the intestinal cicatricial tissue

Animals from the uremia group had significantly lower microvascular density (p=0.0074) than those from the simulation group (Figure 4).

#### *Tissue evaluation of nuclear proliferation nuclear antigen (pcna)*

**FIGURE 5:**
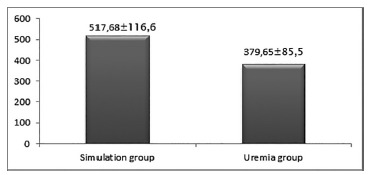
Graph showing the arithmetic averages of cellular proliferation of nuclear antigen quantification in intestinal cicatricial tissue

Uremia-inducing animals demonstrated a significant (p<0.0000) negative impact on cell proliferation in the intestinal cicatricial tissue when analyzed by nuclear proliferation cell antigen expression (Figure 5).

#### *Myofibroblast density evaluation*

**FIGURE 6:**
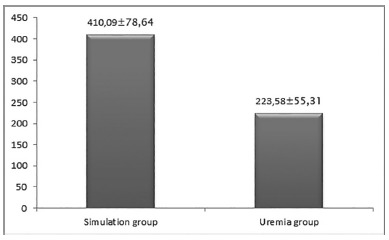
Graph showing the arithmetic means of quantifications of myofibroblasts in the intestinal cicatricial tissue

The quantified density of myofibroblasts in the intestinal cicatricial tissue had a sharp and significant reduction (<0.0001) in animals that had uremia induced in relation to those that did not present with uremia (Figure 6).

## DISCUSSION

Was adopted the experimental model proposed by Vianna et al.^14^ ^,^ ^17^ ^,^ ^33^ with modification. The differential of the present study of this model is the non-accomplishment of ablation of most of the parenchyma opposite the renal wire and the use of argon plasma electrocoagulator for the ablation of 2/3 of renal mass. Experimental studies have demonstrated the superiority of this mode of electrocauterization due to efficiency and limited tissue trauma^13^.

The reproduction of this technique in rats similar to that performed in the present study was performed by Fleck et al^10^, demonstrating a decrease in the glomerular filtration rate after 10 weeks of the procedure, with the particularity of the analysis between genders. The study verified that there was a significant difference between male and female rats, assuming greater fragility and susceptibility in females^10^.

Although other experimental models are also effective in demonstrating renal injury, nephrectomy 5/6 has some advantages such as reproducing renal dysfunction due to loss of nephrons as well as in humans, providing more complete data on proteinuria and hypertension secondary to decreased glomerular filtration, and compare renal damage to the amount of renal mass withdrawn. When performed nephrectomy 1/2, 2/3 and 5/6 analysis of the biochemical and morphological effects of the remaining kidney after progressive renal mass ablation, demonstrates that compensatory renal hypertrophy and glomerular injury are closely related to the volume of the remaining kidney, and therefore, being more evident when a greater fraction of renal tissue is extracted^20^ ^,^ ^29^.

Urea is formed in the liver as the main end product of the metabolism of nitrogenous substances and is subsequently excreted by the kidneys^2^. Experimental and clinical studies have demonstrated the negative effect of uremia on wound healing. Colin et al.^7^ concluded that rats with uremia were delayed in the healing of small intestine anastomoses and aponeurosis. Therefore, the presence of uremic toxins has a negative effect by decreasing cell proliferation, the amount of hydroxyproline in the operative wound, as well as causing alteration in the formation of fibroblasts^7^ ^,^ ^22^.

Uremia can cause intestinal mucosal injury, edema, inflammation, ulceration with loss of mucosal barrier and as a consequence, bacterial translocation may occur, a factor known to be detrimental to the healing process^9^.

Studies have shown that granulation tissue was reduced in uremic mice by performing histological analysis after five days of the procedure for induction of renal dysfunction, as well as inhibition of cell proliferation in fibroblasts and granulation tissue endothelial cells^22^.

To date, 19 isoforms of collagen have been identified, and type I collagen predominates and constitutes 80-90% and the remaining 10-20% are type III^12^. Type I collagen is the most frequent and predominant in bones and tendons is considered mature collagen. Type III is most commonly found in soft tissues such as blood vessels, dermis and fascia. The granulation tissue expresses 30-40% of type III collagen, being considered immature collagen. The most important is type I, which is responsible not only for the maintenance of the integrity of most tissues in function of their mechanical properties^26^, but also for their active participation in the functionality of the tissues as a function of their interaction with the cells present in the matrix extracellular. It is present in all vertebrates, and included in the list of the largest and most complex macromolecules found in the animal kingdom, along with other macromolecules form the extracellular matrix.

Russell et al.^27^, after 10 weeks of nephrectomy and contralateral segmental renal ischemia in rats, demonstrated that there is an evident alteration in the formation of the bone mineral matrix and in the maturation of collagen in the group with uremia.

In a study to evaluate the tensile strength and collagen formation in rats after five, 10 and 15 days of uremia, there was a considerable and significant reduction in collagen accumulation, verified by the quantification of hydroxyproline on the 10^th^ and 15^th^ days^27^.

The production of new vessels is essential in wound repair, and is observed in healing tissues and characterized by a complex process involving multiple stages: vasodilatation, endothelial permeability, rupture of the endothelial cell connection, proliferation and migration of these cells, and subsequent remodeling forming conduits for the passage of nutrients^11^.

Nuclear cell proliferation antigen plays an important role in nucleic acid metabolism. It is a protein synthesized mainly during the S-phase of the cell cycle and is essential for chromosomal chromatin replication, transcription and assembly^8^.

In a study that evaluated the chondrocyte proliferation activity by the expression of cellular proliferation nuclear antigen in the zone of cartilage growth after 30 days of subtotal 5/6 nephrectomy in rats, it was possible to conclude that the number of proliferating cells in the growth plateau was significantly lower in uremic rats when compared to control rats^1^. Another experimental study in rats that investigated the density of myofibroblasts in wound healing by the same method employed in this study concluded that metronidazole, applied topically to wound healing with second intention, does not interfere with the contraction of the wound and delays the appearance of myofibroblasts^32^.

## CONCLUSION

The experimentally induced uremic state is capable of negatively influencing the healing process of the large intestine. Uremia compromises the formation of granulation tissue, delays maturation of type I collagen, induces microvascular density reduction in the anastomosis, reduces cell viability in the area of ​​healing, and density of myofibroblasts present in colonic healing.

## References

[1] Barbosa APF, Silva JDP, Fonseca EC, Lopez PM, Fernandes MBC, Balduino A (2007). Response of the growth plate of uremic rats to human growth
hormone and corticosteroids. Braz J Med Biol Res.

[2] Baum N, Dichoso CC, Carlton CE (1975). Bood urea nitrogen and serum creatinina. Urology.

[3] Becker GJ, Hewitson TD (2013). Animal models of chronic kidney disease useful but not
perfect. Nephrol Dial Transplant.

[4] Boudet J, Man NK, Pils P, Sausse A, Brentano JLF (1978). Experimental chronic renal failure in the rat by
eletrocoagulation of the renal córtex. Kidney Int.

[5] Bravo R Synthesis of the nuclear protein cyclin (PCNA) and its relationship with
DNA.

[6] Brusilow SW, Glew RH, Ninomiya Y (1997). "Inborn errors of urea synthesis". Clinical studies in medical biochemistry.

[7] Colin JF, Elliot P, Ellis H (1979). The effect of uraemia upon wound healing an experimental
study. Br J Surg.

[8] Dou L, Bertrand E, Cerini C, Faure V, Sampol J, Vanholder R, Berland Y, Brunet P The uremic solutes p-cresol and indoxyl sulfate inhibit endothelial
proliferation and wound..

[9] Duarte JBA, Nascimento JEA, Nascimento M, Nochi RJ (2004). Bacterial translocation in experimental uremia. Urol Res.

[10] Fleck C, Appenroth D, Jonas P, Koch M, Kundt G, Nizze H (2006). Suitability of 5/6 nephrectomy (5/6nx) for the induction of
interstitial renal fibrosis in rats - influence of sex, strain, and surgical
procedure. Exp Toxicol Pathol.

[11] Folkman J (1971). Tumor angiogenesis therapeutic implications. N Engl J Med.

[12] Giaquinto MGC, Mota DSC, MARQUES RM (2005). Cicatrização de feridas. Técnica operatória e cirurgia experimental.

[13] Grund KE, Straub T, Farin G (1999). New haemostatic techniques argon plasma
coagulation. Baillieres Best Pract Res Clin Gastroenterol.

[14] Hermann JB, Woodward SC, Pulaski EJ (1964). Healing of colonic anastomosis in the rat. Surg Gynecol Obstet.

[15] Hsu SM, Raine L, Fanger H (1981). Use of avidin-biotin peroxidase complex (ABC) in immunoperoxidase
techniques a comparison between ABC and unlabelled antibody (PAP)
procedures. J Histochem Cytochem.

[16] Iwanaga TC, Aguiar JL, Martins-Filho ED, Kreimer F, Silva-Filho FL, Albuquerque AV (2016). Analysis of biomechanical parameters in colonic
anastomosis. ABCD Arq Bras Cir Dig.

[17] Jiborn H, Ahonen J, Zederfeldt B (1980). Healing of experimental colonic anastomoses III. Collagen
metabolism in the colon after left colon resection. Amer J of Surg.

[18] Junqueira LCU, Montes GS, Sanchez EM (1982). The influence of tissue thickness on the study of collagen by the
picrosirius-polarization method. Histochemistry.

[19] (2002). /DOQI clinical practice guidelines for chronic kidney disease:
evaluation, classification, and stratification. Am J Kidney Dis.

[20] Kaufman JM, DiMeola HJ, Siegel NJ, Lytton B, Kashgarian M, Hayslett JP (1974). Compensatory adaptation of structure and function following
progressive renal ablation. Kidney Int.

[21] Lorena D, Uchio K, Costa AM, Desmoliere A (2002). Normal scarring importance of myofibroblasts. Wound Repair Regen.

[22] McDermott FT, Nayman CM, De Boer WGRM (1968). The effect of acute renal failure upon wound healing histological
and autoradiographic studies in the mouse. Annals of Sur,.

[23] Molgaard HV, Spurr NK, Greaves MF (1989). The hemopoietic stem cell antigen CD34 is encoded by a gene
located on chromosome 1. Leukemia.

[24] Nery RA, Kahlow BS, Skare T, Tabushi FI, Castro A (2015). Uric Acid and Tissue Uric Acid and Tissue Repair. ABCD Arq Bras Cir Dig.

[25] Pincus MR, Henry JB, Henry JB (2008). Química clínica. Diagnósticos Clínicos e tratamento por métodos laboratoriais.

[26] Ramachandran GN, Mitra AK (1976). An explanation for the rare occurrence of cis peptide units in
proteins and polypeptides. J Mol Biol.

[27] Russell JE, Avioli LV (1972). Effect of Experimental Chronic Renal Insufficiency on Bone
Mineral and Collagen Maturation. J Clin Invest.

[28] Salgado FL, Artigiani-Neto R, Lopes-Filho GJ (2016). Growth factors and COX2 in wound healing an experimental study
with Ehrlich tumors. ABCD Arq Bras Cir Dig.

[29] Santos LS, Chin EWK, Ioshii SO, T R (2006). Surgical reduction of the renal mass in rats Morphologic and
functional analysis on the remnant kidney. Acta Cir Bras.

[30] Sesso RC, Lopes AA, Thomé FS, Lugon JR, Watanabe Y, Santos DR (2014). Relatório do Censo Brasileiro de Diálise Crônica. J Bras Nefrol.

[31] Stevens A, Lowe J (2000). Respostas teciduais ao dano. Patologia.

[32] Trindade LCT, Biondo-Simões MLP, Sampaio CPP, Farias RE, Pierin RJ, C M (2010). Avaliação do uso tópico do metronidazol no processo de
cicatrização de feridas um estudo experimental. Rev Col Bras Cir.

[33] Vianna AL, Duarte VT, Araújo RC, Barbosa H (1981). Uremia e cicatrização intestinal estudo experimental em
ratos. Rev Hosp Clin Fac Med S Paulo.

